# A novel optimized pre-embedding antibody-labelling correlative light electron microscopy technique

**DOI:** 10.1099/acmi.0.000750.v3

**Published:** 2024-02-20

**Authors:** Nicole Doyle, Jennifer Simpson, Philippa C. Hawes, Helena J. Maier

**Affiliations:** ^1^​ The Pirbright Institute, Ash Road, Pirbright, Woking, GU24 0NF, UK; ^†^​ Present address: The Francis Crick Institute, Midland Road, London, NW1 1AT, UK

**Keywords:** antibody CLEM, CLEM, confocal microscopy, correlative, coronavirus, CoV, electron microscopy, replication organelle

## Abstract

In the intricate environment of a cell, many studies seek to discover the location of specific events or objects of interest. Advances in microscopy in recent years have allowed for high detail views of specific areas of cells of interest using correlative light electron microscopy (CLEM). While this powerful technique allows for the correlation of a specific area of fluorescence on a confocal microscope with that same area in an electron microscope, it is most often used to study tagged proteins of interest. This method adapts the correlative method for use with antibody labelling. We have shown that some cellular structures are more sensitive than others to this process and that this can be a useful technique for laboratories where tagged proteins or viruses, or dedicated CLEM instruments are not available.

## Data Summary

The authors confirm all supporting data, code and protocols have been provided within the article or through supplementary data files.

## Introduction

Confocal immunofluorescence microscopy techniques are valuable in their ability to reveal large volumes of data on the localization of proteins or events of interest. There is, however, a limit to the level of detail which can be visualized using these techniques. Transmission electron microscopy (TEM) can provide an abundance of ultrastructural detail at a high resolution, but is also a low-throughput technique, only capable of visualizing small areas of interest. For some samples where there are low numbers of events to be visualized, this is a significant problem. Combining these two techniques in correlative light electron microscopy (CLEM) is incredibly powerful, allowing for the correlation of a fluorescent signal from a confocal microscope onto an electron micrograph. It has become an ideal method to attain localization information as well as ultrastructural details of the same event in the same cell at the same time [1, 2]. There are, broadly speaking, two options for CLEM workflows; either the fluorescence and TEM analysis are performed post-embedding on sections, or the fluorescently tagged proteins are imaged pre-embedding and subsequently prepared for room-temperature (RT) TEM analysis. Techniques performed post-embedding often require low temperature stabilization and imaging as well as technically advanced systems such as specific holders, which can be used in both a fluorescent microscope and a transmission electron microscope [2]. This hardware and the expertise needed to use it are only accessible in institutes that have specialized in CLEM. While pre-embedding methods can be more attainable for laboratories with standard equipment, they rely on the availability of a fluorescently tagged protein of interest. In the work presented here, we aimed to adapt CLEM to allow pre-embedding antibody labelling and imaging.

**Fig. 1.**
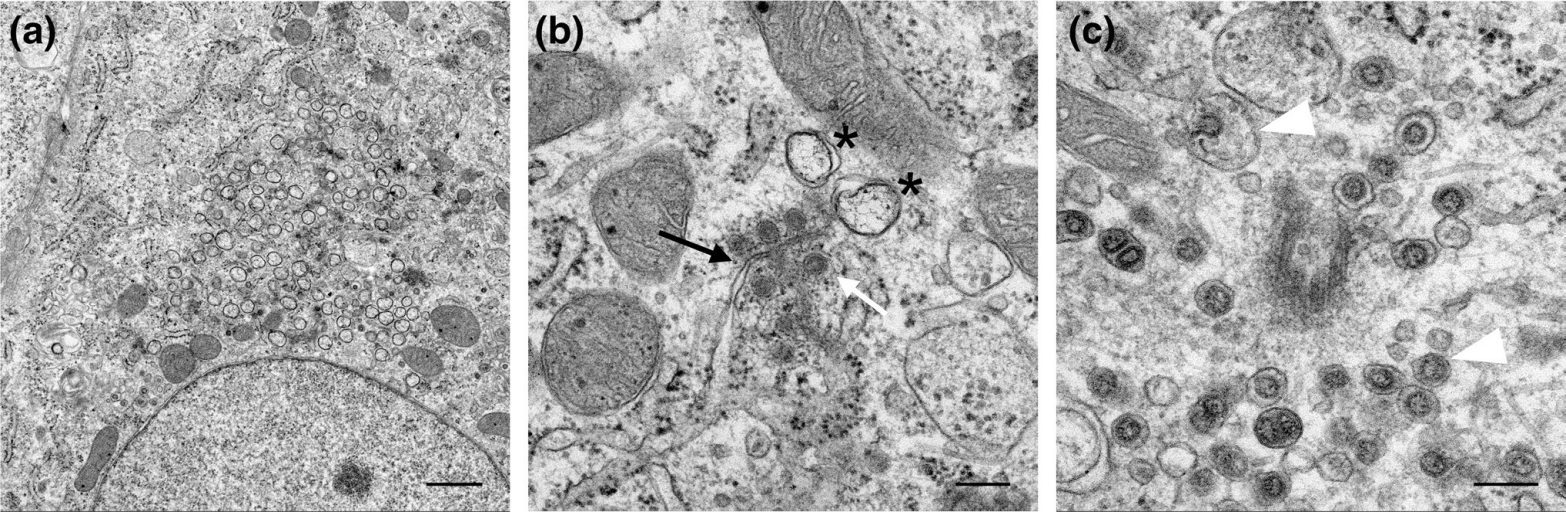
Appearance of IBV replication organelles under standard EM preparation conditions. Primary chick kidney cells were infected with IBV, chemically fixed at 7 h p.i., resin-embedded and sectioned. Double-membrane vesicles (asterisk), spherules (white arrow) and zippered ER (black arrow), as well as virus particles in vesicles (white arrowhead) are indicated. Scale bars indicate 1 µm (**a**) or 200 nm (**b and c**). Source: [5].

Infectious bronchitis virus (IBV) is a *Gammacoronavirus*, which causes disease in poultry, causing substantial economic losses to the poultry industry globally. Our work aims to understand the formation and function of replication organelles (ROs) induced by IBV during infection. These specialized sites provide a platform for the assembly of viral RNA synthesis machinery, as well as a protected environment for RNA synthesis. Coronavirus ROs are comprised of double-membrane vesicles (DMVs) and double-membrane spherules in association with regions of zippered ER or convoluted membranes ([3], Fig. 1). IBV ROs have been found to be consistent in all cell types tested [4, 5]. Coronavirus RNA synthesis is now known to be located to DMVs [3, 6, 7]. Our work aimed to utilize CLEM to visualize the location of nascent viral RNA to DMVs, spherules or zippered ER in IBV-infected chicken cells. A uridine analogue, 5-bromouridine (BrU), which is taken up and used by the cell in the production of new RNA, was used to label nascent viral RNA [6, 8]. We sought to develop a CLEM protocol that would allow detection of this BrU containing RNA using antibodies followed by correlation of fluorescence images with electron micrographs. The protocol was optimized, showing significant improvements in cellular structure to a standard that provides an effective method for some applications [9]. However, some cellular structures appear more susceptible than others to degradation during this protocol and it was ultimately unsuitable for this specific project. We present this CLEM protocol as a potentially useful alternative when fluorescently tagged proteins and specialized equipment are not available for use.

## Methods

### Cells, viruses and media

Avian DF1 cells (LGC Standards) were maintained in Dulbecco’s Modified Eagle’s Medium (DMEM; Merck) supplemented with 10 % foetal bovine serum (FBS; Merck) at 37 °C 5 % CO_2_. IBV (apathogenic strain BeauR [10];) infection of DF1 cells were carried out in 1×BES medium [Modified Eagle's Medium (MEM), 0.3 % tryptose phosphate broth, 0.2 % bovine serum albumin, 20 mM N,N-Bis(2-hydroxyethyl)−2-aminoethanesulfonic acid (BES), 0.21 % sodium bicarbonate, 2 mM l-glutamine, 250 U ml^−1^ nystatin, 100 U ml^−1^ penicillin, and 100 U ml^−1^ streptomycin, Merck]. Porcine LLC-PK1 cells (ATCC CL-101 [11]) were maintained in DMEM supplemented with 10 % FBS. Porcine deltacoronavirus (PDCoV) OH-FD22 was kindly provided by Prof. Linda Saif, The Ohio State University [12, 13]. Viral infection of LLC-PK1 cells was performed in MEM supplemented with 1 % HEPES, 1 % non-essential amino acids, and 1 % antibiotic-antimycotic with 2.5 µg ml^−1^ trypsin (Merck).

### Infection of cells on gridded coverslips

Cells were seeded in Mattek P35H-2–14 C-GRID dishes, which had been sterilized with 70 % ethanol. Once cells reached 70 % confluency, they were washed with PBS and infected. After 1 hour (h), inoculum was removed, the media was changed to IBV or PDCoV infection medium and cells were returned to the incubator until the required timepoint. Thirty minutes (min) before fixing, cells were treated with 5-Bromouridine (BrU; Merck) and actinomycinD (ActD; Merck) as described previously [6]. At the required timepoint, cells were fixed in 4 % paraformaldehyde (PFA, Agar Scientific) for 10 min then immediately labelled.

### Labelling cells on gridded coverslips

All labelling steps for BrU-treated cells were carried out in an RNase-free environment in the presence of RNAsin (Promega), as described previously [6, 8]. Cells were permeabilized in different permeabilization agents as laid out in Table 1 with 0.021 % Triton X-100 finally selected, as described in the results. Cells were then briefly washed in PBS before being incubated in blocking buffer (0.1 % fish gelatin (Merck) in PBS) for 15 min as laid out in the Results. Primary antibodies were diluted in blocking buffer and incubated on cells at room temperature (RT) for 30 min. After washing three times in PBS, cells were incubated for 30 min with AlexaFluor secondary antibodies (ThermoFisher Scientific) in blocking buffer. After washing cells as before, nuclei were labelled with 4′,6-diamidino-2-phenylindole (DAPI; Merck). Cells were briefly rinsed in water then imaged in PBS.

**Table 1.** Permeabilization conditions used in this study

Triton X-100 : 0.1 % in PBS	10 min RT
Saponin: 0.2 % in PBS and blocking buffer	10 min RT
Tween 20 : 0.3 % in PBS	15 min 37 °C
Triton X-100 0.021 % in PBS	10 min RT

### Confocal microscopy imaging

Cells were imaged using a Leica TCS SP8 or Leica STELLARIS 5 laser scanning confocal microscope with inverted stand. To record the location of the region of interest (ROI), a low-magnification brightfield image was taken to capture the entire grid square with grid co-ordinates visible. Higher-magnification images and/or z-stacks were taken of ROI within selected grid squares. Use of the Navigator tool on the Leica STELLARIS 5 software allowed imaging of a larger area without the need to change between microscope objectives to image the ROI. As outlined in the Results, imaging was completed in the shortest timeframe possible to improve cell preservation.

### Fixing, embedding and sectioning cells for electron microscopy

Following collection of fluorescence microscopy images, cells were fixed in 2 % glutaraldehyde for different lengths of time as laid out in the Results. Incubation in 1 % aqueous osmium tetroxide (OsO_4_) solution and 3 % uranyl acetate (UA; Agar Scientific) was optimized as described in the Results, then cells were dehydrated in increasing concentrations of ethanol from 70–100 %. A propylene oxide step was omitted because it reacts with plastic of the Mattek dish. Cells were embedded in Agar 100 epoxy resin according to the manufacturer’s instructions (Agar Scientific). After polymerization at 60 °C, the glass coverslip carefully removed by a brief exposure to liquid nitrogen or a few seconds on a hot plate, and the plastic dish cut away from the resin blocks. Blocks were subsequently trimmed so the identified grid co-ordinates of interest were visible. Sections of 80 nm were cut and collected on hexagonal 200 thin bar copper grids and stained using a Leica EM AC20 prior to imaging.

### Electron microscopy imaging

Samples were visualized and data recorded using either a Tecnai 12 TEM (FEI) at 100 kV with a TVIPS F214 digital camera, or a Talos L120C G2 TEM (ThermoFisher Scientific) at 120kV with a Ceta 4K CMOS camera. Using a low-power image to aid in orientation of the sections, cells of interest selected in confocal microscopy were correlated then the cellular structures investigated. Composite images were compiled using Adobe Photoshop.

## Results

### Optimization of post-fixation conditions to improve preservation of cell structures

**Fig. 2.**
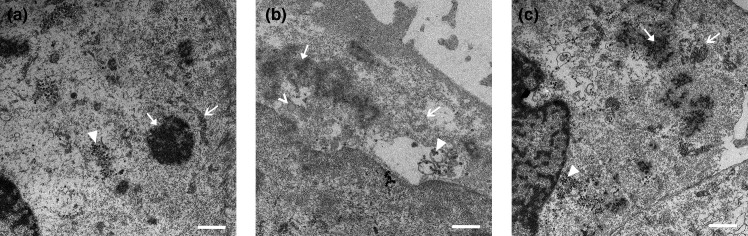
Optimization of fixation improved preservation of cell structures. DF1 cells were infected with IBV, fixed and labelled for immunofluorescence and imaged. Cells were then fixed in EM fix and either incubated in 70 % ethanol (**a**) or osmium tetroxide overnight (**b**). A uranyl acetate incubation between osmium tetroxide and dehydration steps was trialled (**c**). Closed arrows indicate possible spherule patches, closed arrowheads indicate virus particles – either in intact vesicles or loose in the cytoplasm. Empty arrows indicate mitochondria, empty arrowheads indicate lipid droplets. Scale bars indicate 1 µm.

Initial experiments aimed to optimize EM post-fixation steps to maximize preservation of cellular structures. As such, cells were incubated in either 70 % ethanol or osmium tetroxide (OsO_4_) overnight. The longer ethanol incubation was used as a control as this was expected to have a detrimental effect on membranes. The longer osmium incubation provided the potential to improve contrast for EM imaging. As can be seen in Fig. 2a, incubation overnight in ethanol resulted in destruction of most cellular organelle structures; mitochondria (indicated with an empty arrow) were only partially intact and much of the cytoplasmic content was lost. In addition, spherule patches (arrow) were rendered barely recognizable, and while virus particles (arrowhead) were still visible, the membranes which would normally enclose them were lost. By comparison, overnight incubation in OsO_4_ (Fig. 2b) did not have as marked an impact on cell structures with better preservation of intracellular membranes, for example, membranes of vesicles containing virus particles. However, membranes were not always clearly visible under these conditions. Addition of a uranyl acetate (UA) incubation step between the OsO_4_ and ethanol dehydration steps was found to improve the contrast of the images with cellular structures, and particularly the virus-induced spherules appearing more clearly defined (Fig. 2c). Therefore, overnight OsO_4_ incubation combined with a UA incubation step was selected as the condition for subsequent sample processing.

### Cells are sensitive to extended periods in PBS for IF labelling

**Fig. 3.**
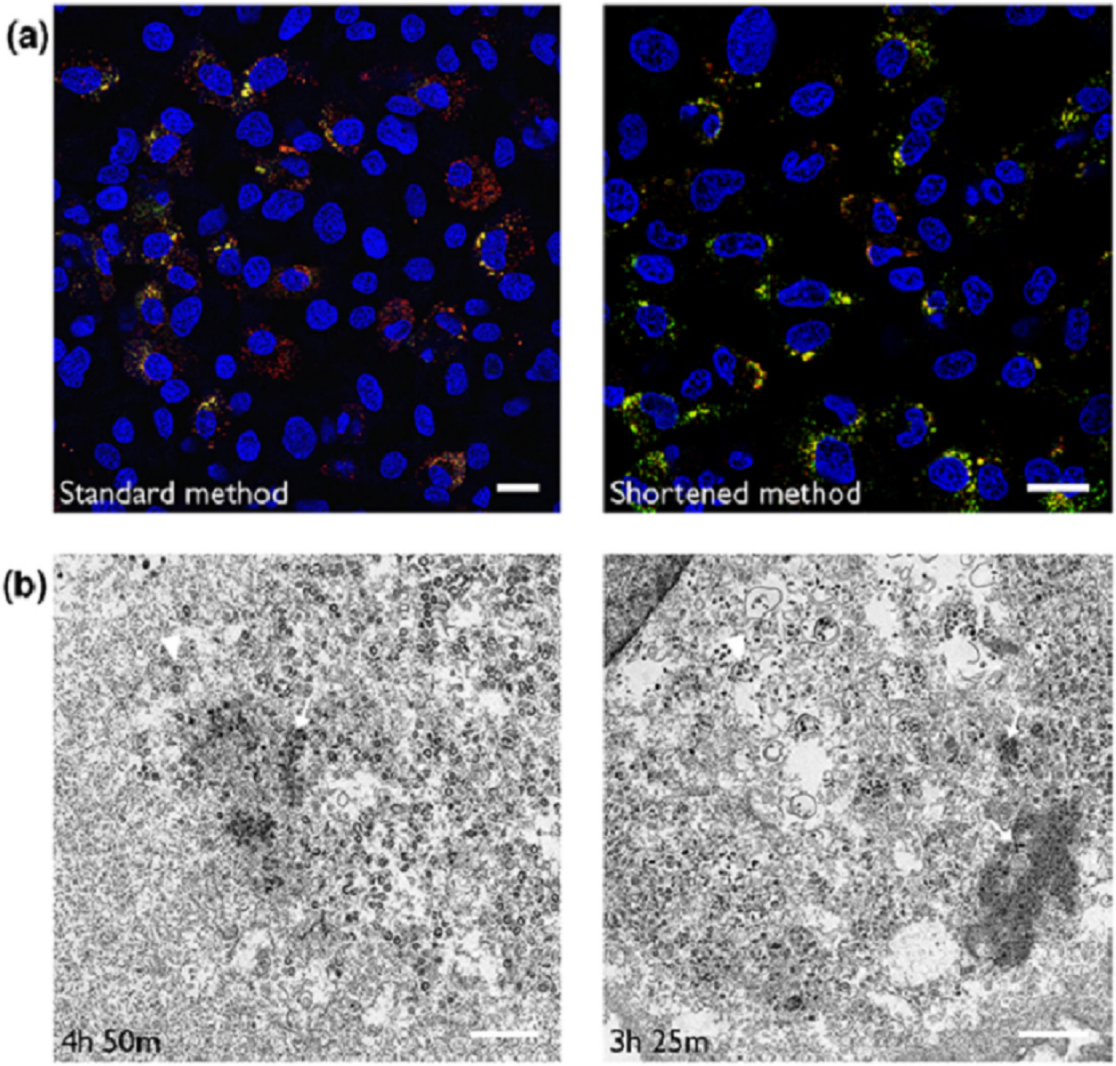
Cells are sensitive to extended periods in PBS during immunofluorescence imaging. (**a**) DF1 cells were infected with IBV, fixed and labelled either using a standard method or a shortened labelling method then imaged by confocal microscopy. Nuclei are stained with DAPI (blue), BrU RNA is shown in green and dsRNA in red. Scale bars indicate 20 µM. (**b**) DF1 cells were infected with IBV, fixed and immunofluorescence labelled then transferred into PBS. Samples were incubated for longer periods of time in PBS for imaging (4 h 50 m), compared with less time in PBS (3 h 25 m). Samples were then fixed and processed for EM. Closed arrows indicate possible spherule patches, closed arrowheads indicate virus particles – either in intact vesicles or loose in the cytoplasm. Scale bars indicate 1 µm.

Although the appearance of the cells was improved using overnight OsO_4_ treatment, cellular structures were still poorly preserved. Due to the non-permanent nature of paraformaldehyde fixation [14] it was considered likely that an extended timeframe for IF labelling was negatively impacting preservation of cells. Therefore, shortening incubation times during the IF labelling protocol was tested. Although standard antibody labelling for immunofluorescence (IF) protocols often state that an hour at RT is required for blocking and both primary and secondary antibody incubations steps, it was found that labelling using a 15 min blocking step followed by 30 min antibody incubation steps resulted in labelling comparable to that using the standard protocol, and this was used for subsequent optimization (Fig. 3a). Furthermore, following completion of IF labelling, the impact of the length of time samples were incubated in PBS during IF imaging prior to further processing for EM was determined. Cells incubated in PBS for a total of 3 h 25 m during IF imaging were compared with those incubated in PBS for 4 h 50 m (Fig. 3b). With longer incubation, the cell structure visualized by EM was more severely compromised with much of the cytoplasmic content being lost and poor preservation of membranes and organelles such as mitochondria. In addition, although virus particles were visible, they appeared free in cytoplasm rather than enclosed in vesicles. Finally, spherule patches detected had an altered morphology to that seen previously. Samples with a shorter incubation in PBS during imaging (Fig. 3b), showed an improvement in preservation of cellular structures. Principally, virus-induced spherules were more visible, although still not complete with intact zER. In addition, the vesicle membrane surrounding virus particles was present in some cases. Therefore, ensuring both rapid IF labelling and the minimum incubation in PBS during imaging prior to EM fixation and embedding is important and was selected for subsequent experiments.

### Effects of permeabilization method on cellular structures

**Fig. 4.**
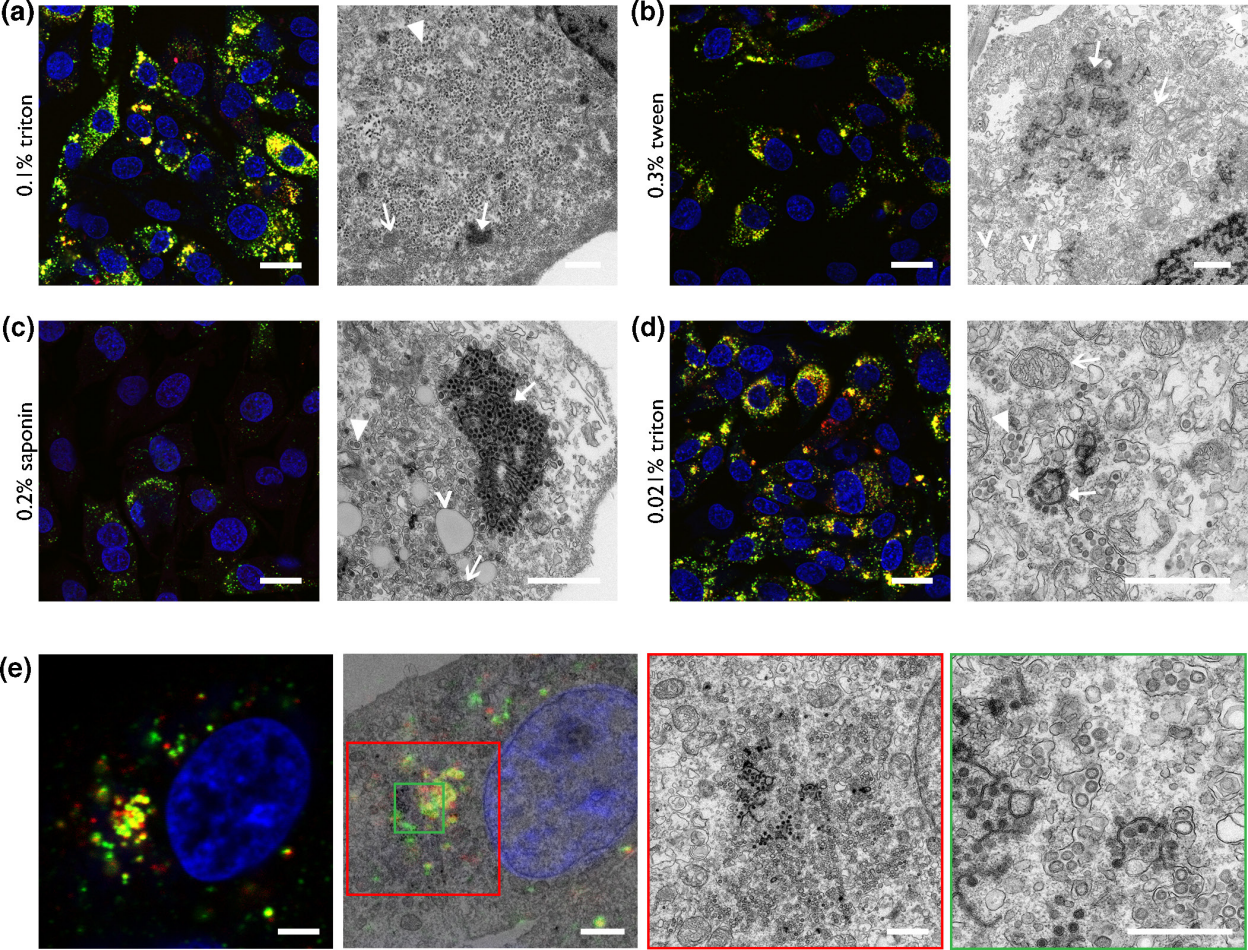
Permeabilization method has an impact on preservation of cellular structures. DF1 cells were infected with IBV, fixed and permeabilised with (**a**) 0.1 % Triton-X 100 (triton), (**b**) 0.3 % Tween20 (tween), (**c**) 0.2 % saponin or (**d**) 0.021 % triton prior to immunofluorescence labelling and confocal imaging or processing for EM. In confocal images, nuclei are stained with DAPI (blue), BrU RNA is shown in green and dsRNA in red. Scale bars indicate 20 µM. In EM images, closed arrows indicate possible spherule patches, closed arrowheads indicate virus particles. Empty arrows indicate mitochondria, empty arrowheads indicate lipid droplets. Scale bars indicate 1 µm. (**e**) Confocal and EM correlated image of IBV in DF1 cells showing intact spherules in the green box and the wider area in the red box. Scale bars indicate 20 µm in confocal and overlay images or 1 µm in EM images.

Optimization of buffer incubation steps had improved the appearance of cellular structures and spherules. However, preservation of cells was still considered inadequate most notably due to the absence of virus-induced DMVs. Therefore, the detergent used for permeabilization during IF labelling was optimized (Table 1). Permeabilization treatment of 0.1 % Triton X-100 for 10 min worked well for IF with high levels of double-stranded RNA (dsRNA) and BrU signals detected. However, it had a significant impact on intracellular structures (Fig. 4a). Mitochondria (indicated by empty arrows) were not clearly visible, and the vesicle membrane surrounding virus particles (closed arrowheads) was missing. Furthermore, spherules (closed arrows) were difficult to recognize. Use of 0.3 % Tween20 for permeabilization was suitable for IF detection of dsRNA and BrU-labelled nascent viral RNA and gave some improvement in overall preservation of cellular and virus-induced structures (Fig. 4b). In particular mitochondria and lipid droplets were now visible. However, these structures still had a somewhat aberrant appearance, presenting with wavy membranes. Use of 0.2 % saponin preserved membrane structures to a much better extent than other permeabilization methods tested, with lipid droplets (empty arrowheads) appearing almost normal, mitochondria present although with wavy membranes, virus particles in vesicles, a large and intact spherule patch detected, alongside potential DMVs. However, saponin resulted in poor detection of BrU (Fig. 4c), making it unsuitable for the specific CLEM experiments here. Finally, lowering the concentration of Triton X-100 was tested. A concentration of 0.021 % was found to be sufficient for IF detection of dsRNA and BrU. In addition, there was improved preservation of the intracellular structures (Fig. 4d), maintaining spherule structures, virus particles and potential DMVs. Although outer mitochondrial membranes were aberrant, the cristae are still intact. In comparison with the images attained in early stages of this process, these conditions were found to best conserve both the detection of BrU and cellular ultrastructure. Furthermore, this protocol allowed for correlation of fluorescence to areas of ROs (Fig. 4e). Unfortunately, however, due to poor preservation of DMVs, conclusions against our research question could not be drawn using this approach.

### Cellular structures appear better preserved in porcine cells

**Fig. 5.**
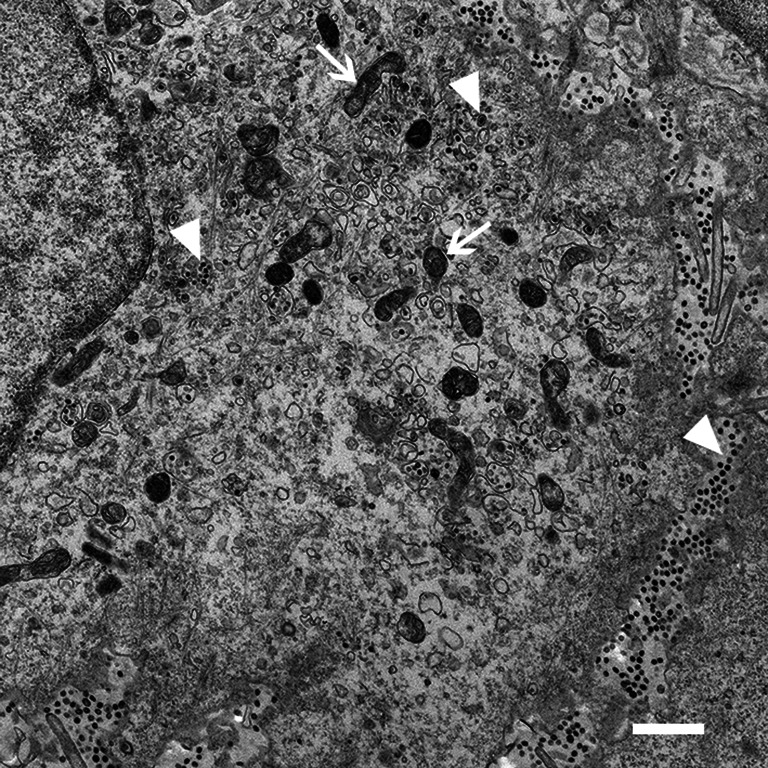
PDCoV-infected porcine LLC-PK1 cells are better preserved during the CLEM protocol. LLC-PK1 cells were infected with PDCoV and then prepared for CLEM using the finalized protocol outlined in the Results. Closed arrowheads indicate virus particles, empty arrows indicate mitochondria. Scale bar indicates 1 µm.

During the course of this work, the applicability of the protocol to study ROs induced by porcine deltacoronavirus in porcine cells was assessed. LLC-PK1 cells were infected with PDCoV and processed using the optimized protocol. Interestingly, cellular structures appeared better preserved than during earlier work with chicken cells (Fig. 5). Mitochondria (indicated with empty arrows) were largely of normal phenotype, although with slightly more wavy membranes than normal, and virus particles could be found within intact vesicles (closed arrowheads). While ROs similar to those seen in IBV-infected cells are usually present in PDCoV-infected LLC-PK1 cells [15], unfortunately, in this instance, ROs were not observed so it was not possible to compare preservation of RO membranes in porcine and chicken cells. However, it would appear that some cell types are more suited to this protocol than others and this should therefore be a consideration during experimental design and optimization.

## Discussion

**Fig. 6.**
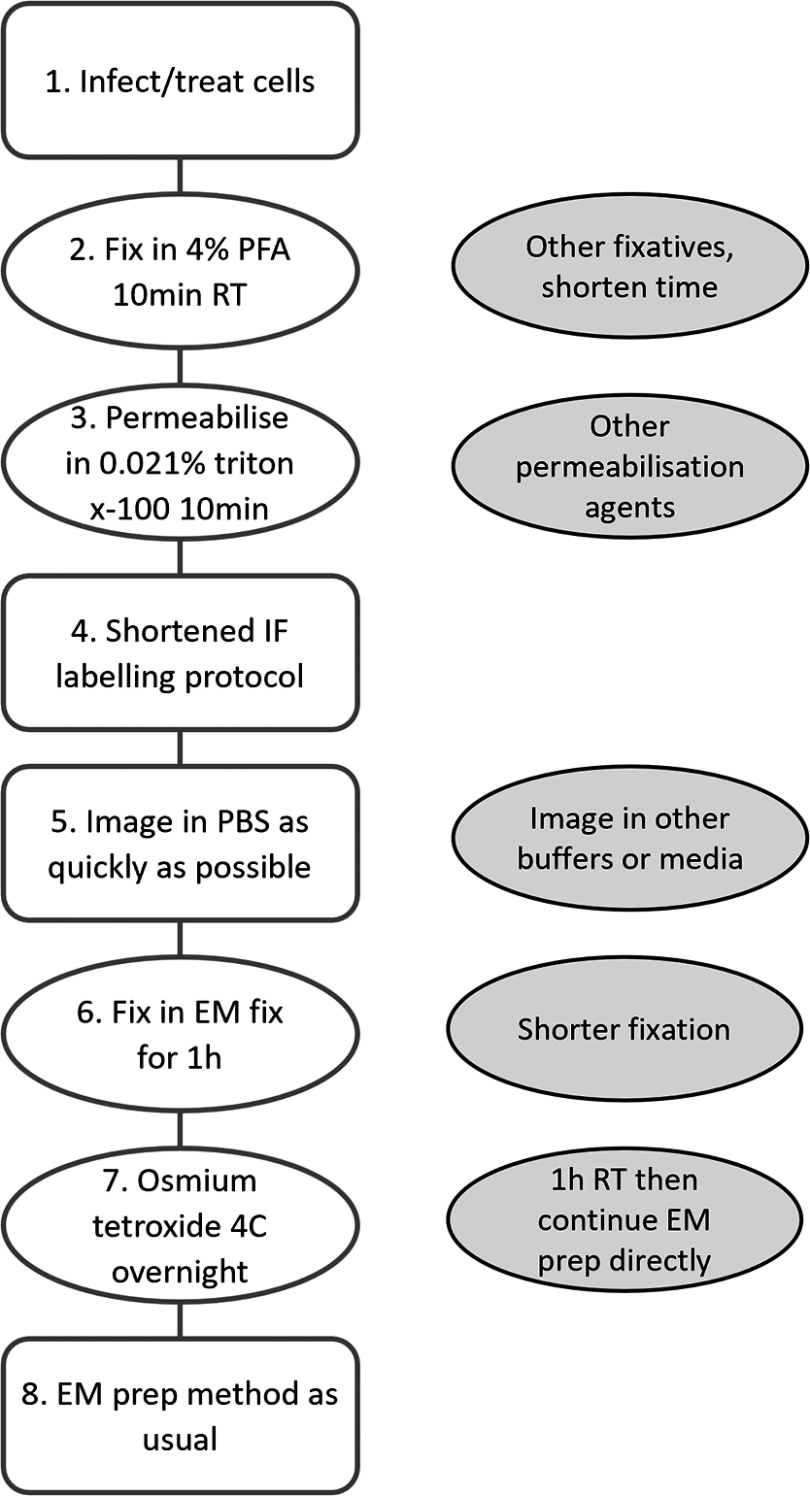
Flowchart of the optimized CLEM preparation method used in this study. After infection, cells were fixed, permeabilized and imaged by IF with EM preparation following as soon as possible thereafter. Points for further optimisation are highlighted in blue circles, corresponding to steps 2, 3, 5, 6 and 7.

CLEM is an adaptable and useful technique, with several variations depending on the biological tools and equipment available (reviewed in [16]). Upon commencing this work, the exact location of IBV RNA synthesis was yet to be elucidated and the hypothesis was that these sites would localize in or around the viral RO, encompassing DMVs and spherules. The aim here was to develop a technique that allowed immunofluorescence labelling of sites of viral RNA synthesis to correlate with ultrastructure imaged by TEM but utilizing only standard confocal and electron microscopes. This is essentially a simple merge of traditional immunofluorescence labelling and imaging with RT chemical fixation and imaging in the TEM with no requirement for specialized equipment or processes. The use of cell-culture dishes with grid co-ordinates engraved on them facilitated easy identification of the ROI imaged by immunofluorescence when later trimming and sectioning the sample embedded in resin for EM. Furthermore, by shortening the immunofluorescence labelling stages, optimizing the detergent used for cell permeabilization and optimizing EM fixation, a protocol was established that allowed correlation of confocal images with EM images (Fig. 6) and there is potential to further optimise the protocol such as by fixing in paraformaldehyde with a low concentration of glutaraldehyde (Fig. 6, point 2), which would stabilize the cell structure but also might interfere with antigens so would depend on the antibody target of interest. Overall, the protocol developed is easily accessible to laboratories that are not CLEM specialists.

Evidently, membrane permeabilization is a key step that impacts cellular integrity. Experimental design here required the use of BrU labelling of nascent viral RNA. This is unstable and highly susceptible to RNases and therefore required specific handling. Furthermore, the DMVs which house the majority of BrU-labelled viral RNA require stringent permeabilization methods to allow antibodies to access their contents [6, 7]. This therefore restricted the use of permeabilization agents. Our earlier work demonstrated that digitonin would not be applicable [6] so was not tested here. It was also found that saponin could not fully permeabilize DMVs. Permeabilization agents used in IF labelling cause perforations in cellular membranes to allow antibodies to enter and label proteins of interest, which consequently compromises membrane integrity. It has been described that permeabilization procedures can have a marked effect on cell structure [17]. Therefore, a balance needs to be struck between adequately permeabilizing membranes while maintaining the integrity of cellular structures. By optimizing permeabilization conditions, the worst effects of these reagents on cell membranes can be avoided. Here, saponin gave the best-preserved samples but it was not suitable for visualising BrU antibody immunofluorescence signal. However, for other research questions, saponin along with digitonin, could provide good alternatives for cell permeabilization (Fig. 6, point 3).

Inherent in this protocol is the requirement for the cells, following IF labelling, to be held in a buffer while collecting fluorescent images. Here, it was demonstrated that extended periods of time in PBS were detrimental to cellular structures. Therefore, ensuring rapid fluorescence imaging is important. There is scope, however, to optimize this stage of the protocol to further minimise impacts on the cell. For example, imaging in different buffers, such as L15, Fluorobrite (ThermoFisher Scientific) or other cell-culture media designed specifically for imaging (Fig. 6, point 5), might improve the appearance of cellular structures and this remains to be tested.

Another interesting observation from this work was that different cell lines appeared to be more or less resilient during the protocol. Porcine LLC-PK1 cells were observed to have markedly improved cell preservation when compared with chicken DF1 cells. It is not clear whether these differences are due to cell species origin or are cell line specific. This would require further investigation. It is clear, however, that compatibility of the cell type with the method needs to be taken into consideration during experimental design.

During establishment of this protocol, it became apparent that cellular structures were not equally impacted by addition of the IF labelling prior to EM sample preparation. Nuclei seemed largely unaffected with both their shape and the nuclear membrane remaining consistent. However, many other cellular organelles were affected to their detriment. Mitochondria were found to be quite readily extracted, showing an altered phenotype in all conditions tested and lipid droplets largely absent or shrivelled under most of conditions. In addition, autophagosomes and lysosomes were not detected in any of the conditions. Finally, holes were occasionally observed in the cytoplasm, indicative of large-scale loss of cellular contents. Virus-related structures were also impacted to a varying degree. Virus particles seemed largely unaffected although the vesicles they are usually enclosed within were more sensitive. Interestingly, spherules appeared less sensitive than DMVs indicating differences in these elements of the RO, which has been suggested before [3, 18]. This may indicate that there are differences in the membranes forming these structures, their lipid composition and potential contents, which might contribute to the differences seen in their survival of this process. This will be interesting to follow up in the future.

Presented here is a pre-embedding antibody CLEM technique which is accessible for non-CLEM specialist laboratories. Unfortunately, the resulting protocol was not ultimately suitable for correlating sites of IBV RNA synthesis with cellular ultrastructure. It has, however, proved useful for other research questions where a membrane-bound structure was not the focus of enquiry [9]. Therefore, offered here is a simple protocol that has the potential to be further adapted and applied to other research questions.
